# Netrin1/DCC signaling promotes neuronal migration in the dorsal spinal cord

**DOI:** 10.1186/s13064-016-0074-x

**Published:** 2016-10-26

**Authors:** Harald J. Junge, Andrea R. Yung, Lisa V. Goodrich, Zhe Chen

**Affiliations:** 1 Department of MCDB, University of Colorado, Boulder, CO 80309 USA; 2 Department of Neurobiology, Harvard Medical School, Boston, MA 02115 USA

**Keywords:** Neuronal migration, Netrin1, DCC, ROBO3, Spinal cord neurons

## Abstract

**Background:**

Newborn neurons often migrate before undergoing final differentiation, extending neurites, and forming synaptic connections. Therefore, neuronal migration is crucial for establishing neural circuitry during development. In the developing spinal cord, neuroprogenitors first undergo radial migration within the ventricular zone. Differentiated neurons continue to migrate tangentially before reaching the final positions. The molecular pathways that regulate these migration processes remain largely unknown. Our previous study suggests that the DCC receptor is important for the migration of the dorsal spinal cord progenitors and interneurons. In this study, we determined the involvement of the Netrin1 ligand and the ROBO3 coreceptor in the migration.

**Results:**

By pulse labeling neuroprogenitors with electroporation, we examined their radial migration in *Netrin1* (*Ntn1)*, *Dcc*, and *Robo3* knockout mice. We found that all three mutants exhibit delayed migration. Furthermore, using immunohistochemistry of the BARHL2 interneuron marker, we found that the mediolateral and dorsoventral migration of differentiated dorsal interneurons is also delayed. Together, our results suggest that Netrin1/DCC signaling induce neuronal migration in the dorsal spinal cord.

**Conclusions:**

Netrin1, DCC, and ROBO3 have been extensively studied for their functions in regulating axon guidance in the spinal commissural interneurons. We reveal that during earlier development of dorsal interneurons including commissural neurons, these molecules play an important role in promoting cell migration.

**Electronic supplementary material:**

The online version of this article (doi:10.1186/s13064-016-0074-x) contains supplementary material, which is available to authorized users.

## Background

During spinal cord development, neuroprogenitors are born in the superficial layer of the ventricular zone (VZ) and undergo radial migration toward the lateral spinal cord [1–3]. Upon neurogenesis, neuroprogenitors exit the cell cycle and migrate out of the VZ. The post-mitotic neurons continue to migrate along both mediolateral and dorsoventral axes as they differentiate into mature neurons. Different classes of interneurons, including contralateral- and ipsilateral-projecting neurons, are generated from progenitors in the dorsal spinal cord. Additional populations of interneurons as well as motor neurons arise from neuroprogenitors in the ventral half. Discrete neuronal populations that are located at different dorsoventral and mediolateral positions have stereotypical axonal projections and specific synaptic partners later during development [1–3]. Given the importance of the migration in building the spinal cord circuitry, it is critical to identify the molecular mechanisms that regulate the radial and tangential migration of spinal cord neurons.

Recently, we found that in the knockout (KO) mice of the *Dcc* (deleted in colorectal carcinoma) receptor, the radial migration of dorsal progenitors and the tangential migration of differentiated dorsal interneurons are both delayed [4]. These migration defects may contribute to the axonal growth and guidance defects in the mutant during later stages of development [4]. In addition, *Dcc* KO has been previously shown to reduce the ventral migration of several classes of dorsal spinal cord interneurons [5]. These findings together prompted us to identify the molecular pathway that DCC acts upon during the migration.

The secreted Netrin proteins are conserved ligands for DCC from *C. elegans* to mammals [6]. Netrin/DCC are important for various neurodevelopmental processes, including axon guidance, neuronal migration, and synapse formation [7–9]. Within the contralateral-projecting commissural axons, Netrin1/DCC signaling induces axonal outgrowth and promotes axonal attraction [10–12]. By immunohistochemistry using specific antibodies, Netrin1 protein has been shown to be enriched at the ventral midline and at the lateral margin of the spinal cord in both chickens and mice [13, 14]. The enrichment of Netrin1 at the ventral spinal cord is consistent with its role in attracting DCC-expressing commissural axons to the midline [13, 14]. The presence of Netrin1 at the dorsal lateral margin confines central axons within the CNS [15], and also inhibits abnormal entry by periphery sensory axons [16]. Whether Netrin1 can also attract the lateral and ventral migration of spinal cord neurons remains unknown.

ROBO3 is a member of the ROBO (roundabout) family of receptors for the SLIT proteins. While mammalian ROBO1 and ROBO2 mediate repulsion in commissural axons, ROBO3 inhibits ROBO1/2 and thus represses repulsion as commissural axons approach the midline [17]. In addition, ROBO3 has been shown to interact with DCC and function as a Netrin1 coreceptor to potentiate commissural axonal outgrowth and attraction [18]. Thus, ROBO3 is critically important for commissural axon guidance. We wondered if ROBO3 is also involved in the earlier migration process.

In this study, we examined neuronal migration in *Ntn1* and *Robo3* KOs*,* in direct comparison with *Dcc* mutants. Using pulse labeling of the dorsal spinal cord progenitors, we found that the radial migration of these cells is delayed in *Ntn1* and *Robo3* KOs, as found previously in *Dcc* KOs. In addition, using immunohistochemistry of interneuron markers, we found that the tangential migration of dorsal interneurons is also delayed in all three mutants. Our data suggest that the Netrin1 ligand functions through DCC and ROBO3 receptors to promote the migration of the dorsal spinal cord neurons.

## Methods

### Mice

*Ntn1, Dcc, Robo3,* and *Unc5c* KOs were generated and described previously [19–22].

### Whole embryo culture

The culture was carried out as previously described [23]. Embryos were electroporated at E9.5 with *gfp* into one side of the spinal cord and were cultured for specified periods. The embryos were then fixed in 4 % paraformaldehyde, cryopreserved in 30 % sucrose, and embedded in OCT (optimal cutting temperature). 20 μm transverse sections were collected and examined using fluorescent microscopy.

### Immunohistochemistry

Immunohistochemistry was carried out as previously descried [24]. Antibodies used in the study include anti-PAX3/7 (PA1-107, Thermo Fisher, raised against PAX3 and cross reacts with PAX7), anti-BARHL2 (NBP2-32013, Novus Biologicals), anti-LHX5 (AF6290, R&D), anti-ISL1/2 (39.4D5, DSHB), anti-PAX6 (DSHB), anti-phospho-Histone H3 (9701, CST), anti-Ki67 (12202, CST), and anti-SOX2 (3728, CST).

### Quantification of phenotypes

For phenotypic quantification in all experiments, KOs were compared with WT littermate controls. To minimize developmental variation, we used embryos of comparable sizes and examined spinal cord tissues from the brachial level. At least three embryos for each genotype and at least five sections from each embryo were quantified. In all phenotypic analyses, the defects were consistently seen in all embryos examined. The severity of defects was comparable between animals and between different sections of the same embryo. Representative images are shown in all figures.

For quantifying the ventral migration of interneurons, the distance from the dorsal margin of the spinal cord to the ventral most BARHL2+ neurons (h1) is compared to the total height of the spinal cord (h2) (Figs. 6, 7). For the lateral migration, the distance between the medial most and lateral most BARHL2+ neurons (w1) is compared to the distance between the medial most neurons and the lateral margin of the spinal cord (w2). The distances were measured using ImageJ (NIH, Bethesda, MD).

## Results

**Fig. 1:**
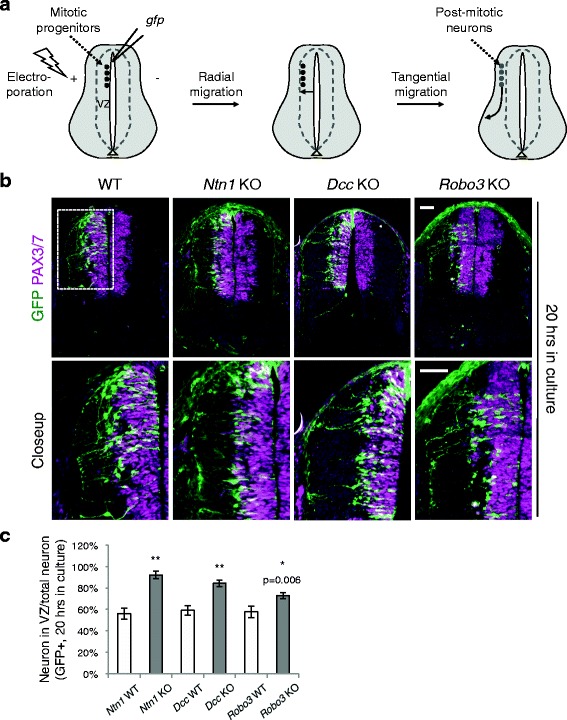
Neuroprogenitor migration in *Ntn1*, *Dcc*, and *Robo3* knockouts. **a** Schematic of the migration of the dorsal spinal cord progenitors and interneurons. VZ, ventricular zone. **b** Cross sections of the spinal cord electroporated with *Actb-gfp* (*βactin-gfp)*. The closeup images are of the boxed area. The embryos were cultured for 20 h. GFP+ neurons from all three KOs migrate out of the VZ (demarcated by PAX3/7 staining) later than WT neurons. **c** Quantification of the ratio between GFP+ neurons within the VZ and the total GFP+ neurons. A higher percentage of neurons is seen within the VZ in all three KOs. Data are represented as the mean ± SEM (Student’s *t*-test; **, *p <* 0.001; *, *p <* 0.05). Scale bars, 50 μm

**Fig. 2:**
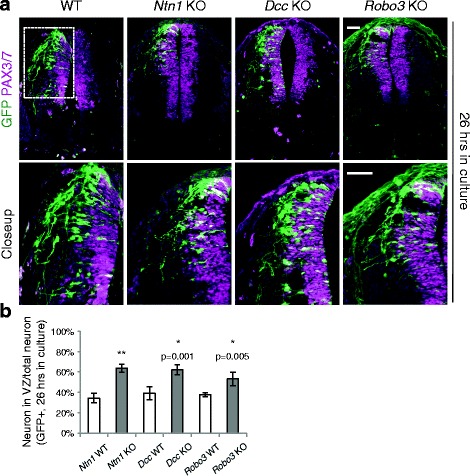
Neuroprogenitor migration in *Ntn1*, *Dcc*, and *Robo3* knockouts. **a** Cross sections of the spinal cord electroporated with *Actb-gfp* (*βactin-gfp)*. The closeup images are of the boxed area. The embryos were cultured for 26 h. Some GFP+ neurons from the KOs are able to exit the VZ (PAX3/7+ area) after 26 h. **b** Quantification of the ratio between GFP+ neurons within the VZ and the total GFP+ neurons. A higher percentage of GFP+ neurons is seen within the VZ in the KOs than in controls. Data are represented as the mean ± SEM (Student’s *t*-test; **, *p <* 0.001; *, *p <* 0.05). Scale bars, 50 μm

By microinjection and electroporation, we introduced *Actb-gfp* (aka *βactin-gfp)* into the progenitors adjacent to the lumen of the neural tube at E9.5 (Fig. 1a). During the subsequent culturing of the whole mouse embryos, the mitotic progenitors first migrated laterally away from the ventricle and can continue to divide [1–3]. At the onset of neurogenesis around E10 in mice, the progenitors began to exit the cell cycle and to migrate out of the VZ. After culturing, we labeled the VZ with antibodies against PAX3/7, which are expressed by the dorsal progenitors [1, 2], and examined the lateral movement of the GFP+ progenitors. In this assay, we previously observed that the dorsal progenitors are more frequently targeted than the ventral ones [4, 23], likely due to the fact that the dorsal progenitors are more actively proliferating and differentiating during the culture period. We first cultured the embryos for 20 h and examined the positions of GFP+ neurons. In WT embryos, many progenitors have exited the VZ and reached the lateral spinal cord. However, in *Dcc* KOs, the majority of GFP+ neurons were still positioned within the VZ ([4], Fig. 1b,c). Similarly, GFP+ progenitors in *Ntn1* and *Robo3* KOs were also mostly found within the VZ (Fig. 1b,c). We extended the culture period to 26 h to determine if the mutant progenitors are able to exit the VZ later. After the longer culture period, most WT neurons have exited the VZ and have extended axons toward the ventral half (Fig. 2). In comparison, neuroprogenitors in all three mutants were able to arrive at the lateral spinal cord, but the percentage of neurons within the VZ was still higher than in WT (Fig. 2). These results suggest that the radial migration of neuroprogenitors is likely to be delayed, but not completely blocked, in *Ntn1*, *Dcc*, and *Robo3* KOs.

**Fig. 3:**
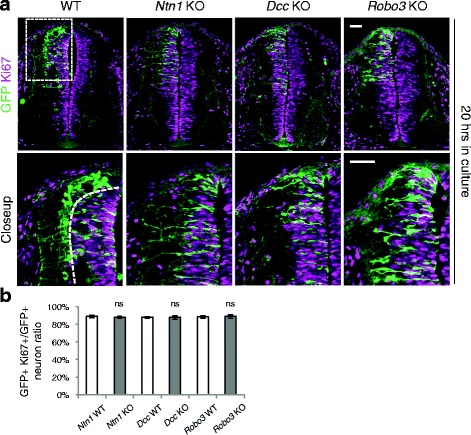
Cell proliferation state in neuroprogenitors in *Ntn1*, *Dcc*, and *Robo3* knockouts. **a** Cross sections of the spinal cord from embryos cultured for 20 h. The closeup images are of the boxed area and the dashed line outlines the VZ. Anti-Ki67 staining marks neurons that are inside the cell cycle. **b** Quantification of the ratio between GFP+ Ki67+ neurons and the total GFP+ neurons within the VZ. The percentage of neurons that are inside the cell cycle is comparable between all three KOs and WT controls. Data are represented as the mean ± SEM (Student’s *t*-test; ns, not significant). Scale bars, 50 μm

As the exit of neuroprogenitors from the VZ and their leaving the cell cycle are highly coordinated, the delay in reaching the lateral spinal cord can also result from a cell cycle defect. To examine this possibility, we studied the GFP+ neurons within the VZ using the Ki67 cell proliferation marker. We found that the percentage of neurons that are proliferating (i.e. Ki67+) is comparable between the three mutants and their respective controls (Fig. 3). These results suggest that the increase in GFP+ neuroprogenitors within the VZ is unlikely to result from a cell cycle abnormality.

**Fig. 4:**
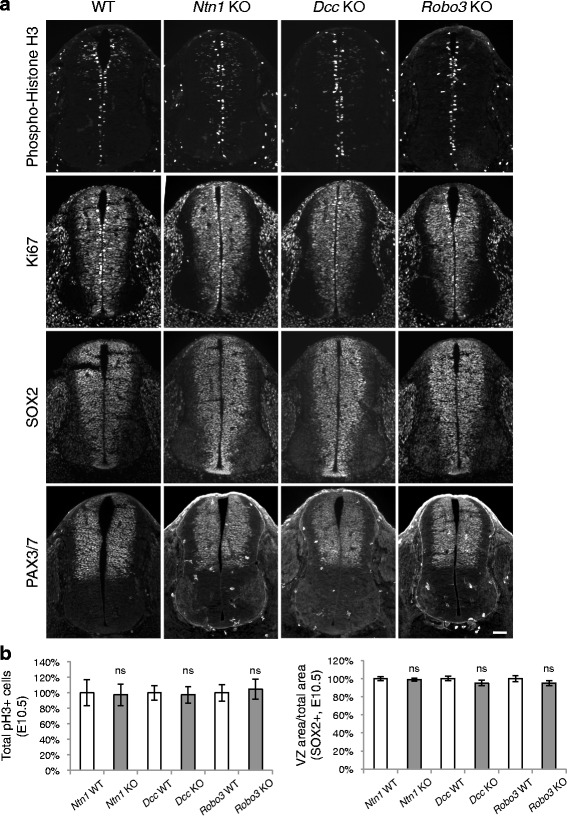
Neuroprogenitors are generated normally in *Ntn1*, *Dcc*, and *Robo3* knockouts. **a** Immunohistochemistry of phospho-Histone H3, a mitotic marker, Ki67, a cell proliferation marker, SOX2, a neuroprogenitor marker, and PAX3/7, a dorsal progenitor marker in E10.5 spinal cord. **b** Quantification of phenotypes in (a). Data are normalized to WT and are represented as the mean ± SEM (Student’s *t*-test; ns, not significant). Scale bar, 50 μm

Previously, we found that in *Dcc* KOs, the overall development of neuroprogenitors, except for their migration, is unaffected [4]. Using the same approaches, we further examined *Ntn1* and *Robo3* KOs. We analyzed cell proliferation using cell cycle markers, including phospho-Histone H3 (pH3), a mitosis-specific marker, and Ki67. We found that the number of neural stem cells and progenitors is normal in *Ntn1* and *Robo3* mutants (Fig. 4). In addition, we labeled neuroprogenitors in the whole spinal cord with anti-SOX2 and the dorsal progenitors with anti-PAX3/7, and found no change in the localization or organization of these progenitor populations (Fig. 4). Thus, stem cells and progenitors are generated normally in the mutants.

**Fig. 5:**
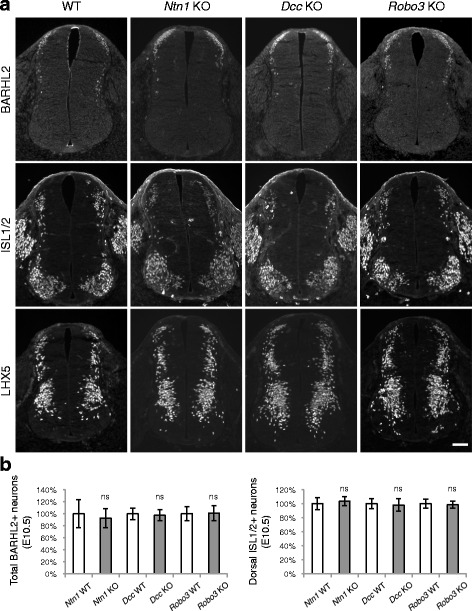
Neuronal differentiation is normal in *Ntn1, Dcc*, and *Robo3* knockouts. **a** Immunohistochemistry of BARHL2, ISL1/2, and LHX5, in E10.5 spinal cord. The markers are expressed by different populations of interneurons and motor neurons. **b** Quantification of BARHL2+ neurons and ISL1/2+ dorsal interneurons. Data are normalized to WT and are represented as the mean ± SEM (Student’s *t*-test; ns, not significant). Scale bar, 50 μm

We also examined if the differentiation of progenitors into interneurons and motor neurons is normal. Using the BARHL2, ISL1/2, and LHX5 markers, which are transcription factors expressed by different populations of neurons [1–3], we found that a normal number of neurons are born in *Dcc* KOs at E10.5 [4]. Using the same assays, we examined *Ntn1* and *Robo3* KOs and found that neither KO displayed any abnormalities in neuronal differentiation (Fig. 5). *Ntn1* KOs were also reported to have a normal number of spinal cord neurons at E11.5 and E13.5 [21, 25]. Therefore, the loss of *Ntn1*, *Dcc,* or *Robo3* does not affect neurogenesis, either.

**Fig. 6:**
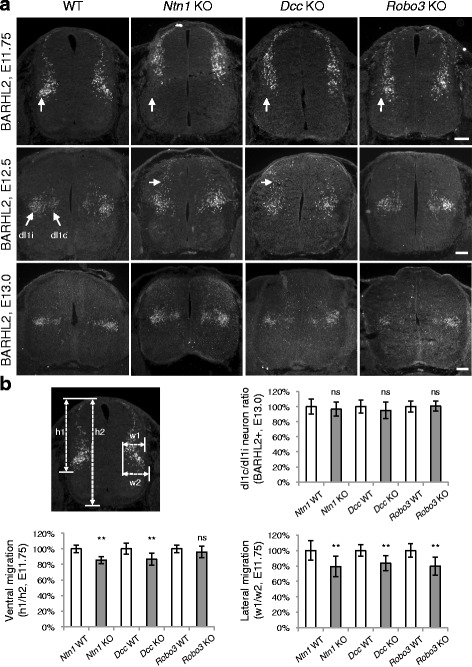
Dorsal interneuron migration is delayed in *Ntn1, Dcc*, and *Robo3* knockouts. **a** Immunohistochemistry of BARHL2 at different stages. At E11.75, BARHL2+ neurons fail to reach the lateral spinal cord in the KOs (indicated by arrow). Their ventral migration is also delayed. At E12.5, dI1i (ipsilateral) and dI1c (contralateral) populations appear normal in WT and *Robo3* KOs. There are still some neurons in the dorsal spinal cord in *Ntn1* and *Dcc* KOs (arrow). At E13.0, all mutants have two distinct populations of dI1 neurons. **b** Quantification of phenotypes in (a). For quantifying the ventral migration, the distance from the dorsal margin to the ventral most BARHL2+ neurons (h1) is compared to the total height of the spinal cord (h2). For the lateral migration, the distance between the medial most and lateral most BARHL2+ neurons (w1) is compared to the distance between the medial most neurons and the lateral margin of the spinal cord (w2). The distances were measured using ImageJ. Data are normalized to WT and are represented as the mean ± SEM (Student’s *t*-test; **, *p <* 0.001; ns, not significant). Scale bars, 50 μm

Differentiated spinal cord neurons continue to migrate along mediolateral and dorsoventral axes to reach the final positions [1–3]. The dI1 population of interneurons are born at the dorsal margin of the spinal cord. They migrate both laterally and ventrally and give rise to contralateral- and ipsilateral-projecting subpopulations (dI1c and dI1i, respectively). Using anti-BARHL2 to follow the tangential migration of dI1 neurons [26], we previously found that both mediolateral and dorsoventral migration is delayed in *Dcc* KOs [4]. We further examined *Ntn1* and *Robo3* KOs. As discussed above, a normal number of BARHL2+ neurons are born at E10.5 in the mutants (Fig. 5). By E11.75, most neurons in WT have migrated ventrally and some start to arrive at the lateral margin of the spinal cord ([26], Fig. 6a). In contrast, in *Ntn1, Dcc,* and *Robo3* KOs, BARHL2+ neurons were located more medially and dorsally than normal (Fig. 6). Later at E12.5, the dI1c and dI1i populations can be well discerned in WT, with dI1i residing at a more lateral position ([26], Fig. 6a). Similarly, *Robo3* KOs had two distinct subpopulations (Fig. 6). *Ntn1* and *Dcc* KO neurons appeared to be slightly more medially and dorsally positioned. At E13.0, dI1c and dI1i could be seen in all three mutants and the ratio between them was normal (Fig. 6). Therefore, there is a delay in the tangential migration of dorsal interneurons in all three mutants, but both contralateral and ipsilateral neurons are eventually differentiated. The delay is less severe in *Robo3* KOs than in *Dcc* KOs, consistent with previous observations in commissural axonal outgrowth [18]. *Ntn1* KO, on the other hand, caused a more severe defect than *Dcc* KO, also consistent with previous observations in axon guidance [21, 25]. This is likely due to the fact that another homologue of DCC, the Neogenin (Neo1) receptor, has some redundant function and thus could compensate for the loss of DCC [24].

**Fig. 7:**
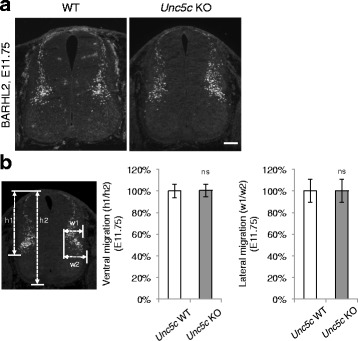
dI1 interneuron migration is normal in *Unc5c* knockouts. **a** BARHL2+ immunohistochemistry in WT and *Unc5c* KO spinal cord at E11.75. The dorsal interneurons have arrived at the normal positions. **b** Quantification of neuronal migration (see Fig. 6 for description of quantification). Data are normalized to WT and are represented as the mean ± SEM (Student’s *t*-test; ns, not significant). Scale bar, 50 μm

The UNC5 family of receptors also bind Netrin and can act as DCC coreceptors. UNC5s by themselves or in complex with DCC mediate axonal repulsion [9]. UNC5s have not been shown to function in spinal commissural axons. In addition, *Ntn1* null mutants display rather distinct phenotypes from the knockout of the *Unc5* family receptors, suggesting that Netrin1 is not the main ligand for UNC5s in vivo [21]. For comparison, we also examined *Unc5c* KOs for the migration phenotype using the BARHL2 marker. At E11.75, when *Ntn1*, *Dcc*, and *Robo3* KOs displayed a migration delay, *Unc5c* KOs did not have such a defect and the BARHL2+ neurons have reached comparable positions as in WT controls (Fig. 7). Taken together, the UNC5 repulsive receptors are unlikely to be involved in the same migration process as Netrin1 and DCC.

By in situ hybridization and immunohistochemistry, *Dcc* expression is observed in interneurons and motor neurons, as well as in neuroprogenitors [4, 5, 27, 28]. *Robo3* expression is more specific and mostly within commissural interneurons [20, 23]. Using specific antibodies, we found that ROBO3 protein is also present on the periphery of dorsal progenitors (Additional file 1: Figure S1). Therefore, *Dcc* and *Robo3* are most likely to function cell-autonomously within progenitors and interneurons during migration, as they do during axon guidance.

## Discussion

**Fig. 8:**
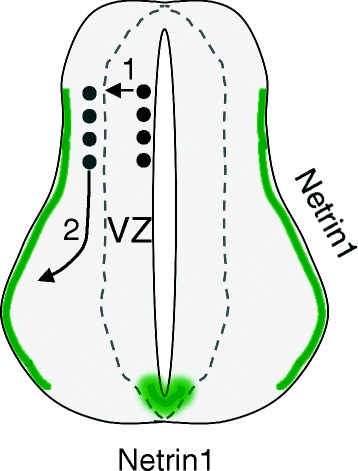
Model of Netrin1/DCC signaling during neuronal migration in the dorsal spinal cord. Netrin1 protein is enriched at the ventral midline and at the lateral margin of the spinal cord (green areas). It acts through DCC and ROBO3 receptors to attract the neurons both laterally and ventrally. 1, radial migration of neuroprogenitors; 2, tangential migration of dorsal interneurons; VZ, ventricular zone

Neuronal migration is one of the early and critical steps of neural development. It is a complex cellular process involving many classes of molecules, including extracelluar ligands and transmembrane receptors, intracellular signaling molecules, cytoskeletal and motor proteins, and transcriptional factors [29–32]. Within the developing spinal cord, the molecular mechanism underlying the migration of neuroprogenitors and differentiated neurons is mostly uncharacterized. The Reelin/VLDLR/ApoER2 pathway has been shown to regulate the migration of certain populations in the ventral spinal cord [33]. Our previous study reveals that DCC is important for the migration of the dorsal neuroprogenitors and interneurons [4]. DCC has also been shown to promote the ventral migration of several classes of dorsal spinal cord interneurons [5]. Built upon these findings, we extended the study to the Netrin1 ligand and the ROBO3 coreceptor, which act together with DCC to mediate axonal attraction. Interestingly, the loss of any of these three molecules reduces the radial migration of neuroprogenitors and the tangential migration of dorsal interneurons. In contrast, loss of the UNC5C receptor does not cause such a delay. Given the enrichment of Netrin1 at the lateral and ventral margin of the spinal cord [13, 14], our results support a model that Netrin1/DCC signaling also attracts the migration of the dorsal spinal cord progenitors and neurons (Fig. 8).

Our experimental approach does not allow us to efficiently target the ventral spinal cord progenitors in cultured mouse embryos. We thus cannot determine if Netrin1/DCC also acts in the ventral populations. Within the ventrally located motor neurons, Netrin1/DCC as well as the SLIT/ROBO pathway have been shown to be important for the dorsoventral positioning of the cell bodies relative to the midline [34]. The loss of the Netrin1/DCC signaling leads to motor neurons positioning in a more dorsal position than normal [34]. Therefore, Netrin1/DCC is likely to be involved in the migration of additional spinal cord neurons. *Robo3* is not expressed in the ventral spinal cord, except in the ventral most V3 interneurons [20, 23], and is thus unlikely to play a role in most ventral neurons.

The fact that in *Ntn1*, *Dcc*, and *Robo3* KOs, neuronal migration is delayed but not completely blocked suggests that there may be additional molecules at play. In addition, although the early-born dorsal interneurons (generated from E10 to around E11.5) migrate predominantly ventrally, late-born populations (generated around E12) migrate dorsally [1, 2]. It is thus likely that a distinct pathway directs the dorsal migration. Additional studies are warranted to unveil the identity of these molecules.

## Additional file

Additional file 1: Figure S1. ROBO3 expression in the spinal cord. ROBO3 is highly expressed in differentiated commissural neurons and their axons, and can also be detected at the periphery of progenitors. PAX7 labels the dorsal progenitors. PAX6 labels progenitors except in the ventral most spinal cord. Scale bars, 50 μm in the top panel and 10 μm in the bottom. (PDF 7076 kb)

## Abbreviations

ApoER2: Apolipoprotein E receptor 2
DCC: Deleted in colorectal carcinoma
KO: Knockout
Ntn1: Netrin1
Robo: Roundabout
Unc: Uncoordinated
VLDLR: Very low density lipoprotein receptor
VZ: Ventricular zone
WT: Wild type
