# Supplementary figures and images for: Netrin1/DCC signaling promotes neuronal migration in the dorsal spinal cord
Source: Neural Dev. 2016 Oct 26;11:19. doi: 10.1186/s13064-016-0074-x (PMC5081974; doi:10.1186/s13064-016-0074-x)

Wildtype, E10.5

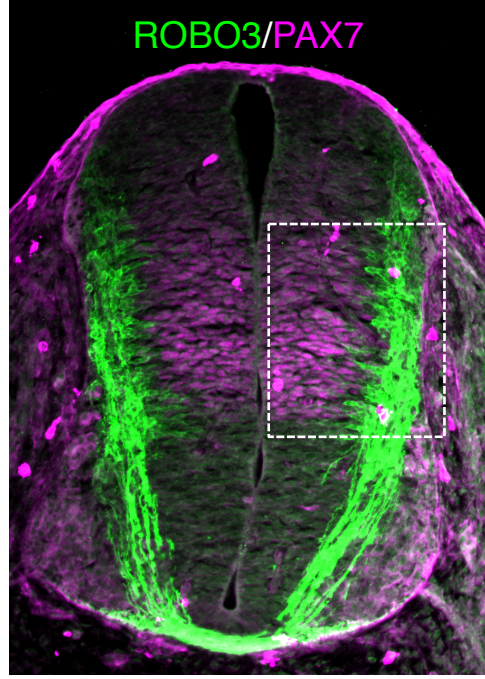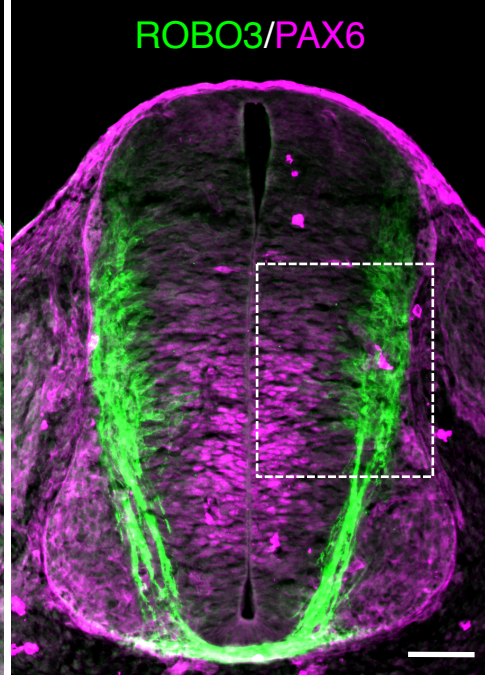

Closeup

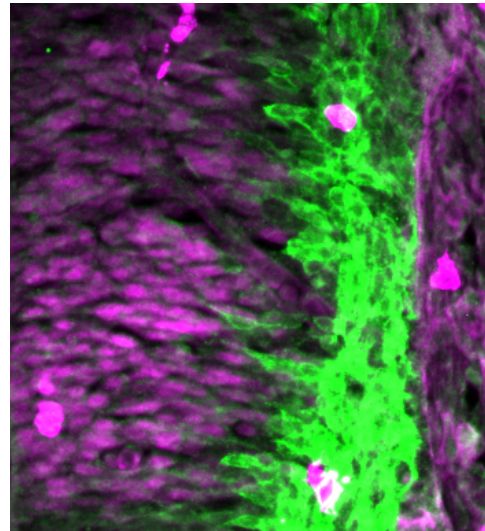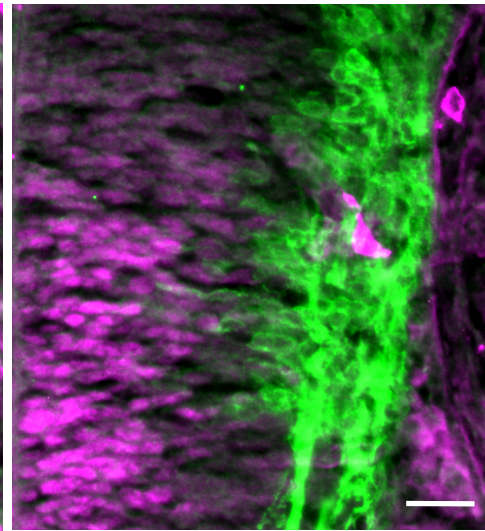

Supplement: Additional file 1: Figure S1. — ROBO3 expression in the spinal cord. ROBO3 is highly expressed in differentiated commissural neurons and their axons, and can also be detected at the periphery of progenitors. PAX7 labels the dorsal progenitors. PAX6 labels progenitors except in the ventral most spinal cord. Scale bars, 50 μm in the top panel and 10 μm in the bottom. (PDF 7076 kb) [file 13064_2016_74_MOESM1_ESM.pdf]
